# Enhanced pedestrian trajectory prediction via overlapping field-of-view domains and integrated Kolmogorov-Arnold networks

**DOI:** 10.1371/journal.pone.0322722

**Published:** 2025-06-09

**Authors:** Hongxia Wang, Yang Liu, Zhenkai Nie

**Affiliations:** School of Information Science and Engineering, Shenyang Ligong University, Shenyang, Liaoning, China; Tongji University, CHINA

## Abstract

Accurate pedestrian trajectory prediction is crucial for applications such as autonomous driving and crowd surveillance. This paper proposes the OV-SKTGCNN model, an enhancement to the Social-STGCNN model, aimed at addressing its low prediction accuracy and limitations in dealing with forces between pedestrians. By rigorously dividing monocular and binocular overlapping visual regions and utilizing different influence factors, the model pedestrian interactions more realistically. The Kolmogorov-Arnold Networks (KANs) combined with Temporal Convolutional Networks (TCNs) greatly improve the ability to extract temporal features. Experimental results on the ETH and UCY datasets demonstrate that the model reduces the Final Displacement Error (FDE) by an average of 23% and the Average Displacement Error (ADE) by 18% compared to Social-STGCNN. The proposed OV-SKTGCNN model demonstrates improved prediction accuracy and better captures the subtleties of pedestrian movements.

## Introduction

A significant area of AI technology is intelligent transportation, which encompasses a wide range of application scenarios, such as intelligent video surveillance [1, 2], autonomous vehicles [3–5], robot navigation [6–8] and other [9]. One essential component is pedestrian trajectory prediction. Additionally, it has received more and more attention lately because of its growing relevance in applications. Predicting pedestrian movements is a challenging task, though. First and foremost, given their vulnerability, small stature, and abundance, we must address pedestrian safety, the main objective of research efforts in areas like autonomous driving. As a result, increasing the precision of pedestrian trajectory prediction and preventing collisions in line with actual circumstances are essential for safely operating some automated devices in intelligent transportation. Secondly, multi-agent trajectory forecasting is very challenging due to the intricate social interactions among pedestrians, where one pedestrian’s actions can influence others. Because pedestrians are highly subjective and can change their speed and direction of movement at any time—especially when their intended path and destination are unknown—using them as research subjects would increase randomness and uncertainty.

The remedy to pedestrian trajectory prediction problems has evolved from physical models to deep learning-based data-driven models. The first strategy for pedestrian behavior modeling that was put forth and put into practice was the social force model [10], which describes how pedestrians interact using energy potential fields. Such physics-based models have been explored extensively and emerged as a significant method for addressing pedestrian trajectory issues. In recent years, the public has gradually moved away from physics model-based approaches in favor of the data-driven approach to modeling pedestrian behavior. The Social-LSTM [11] neural network design, a representative algorithm of the RNN basic model, is one of the most significant neural network architectures in pedestrian trajectory prediction. The approach overemphasized the most critical interactions in the scene, thereby ignoring other interactions. It uses a pooling mechanism to aggregate many features to simulate the social interactions of pedestrians and expects unique hidden states to capture the movement characteristics of pedestrians. The modeling of trajectories and local-global interactions is implemented by LG-LSTM [12]; however, because of its set grid layout, it is not adaptable to various scenarios. Trajectory prediction networks relying on basic LSTM sequence models can yield accurate results. Still, their prediction outputs are only single predicted trajectories closely suited to the dataset findings and lack dynamism and stochasticity. Numerous prediction techniques based on generative models have been presented to address this issue. Using the max-pooling method, Social-GAN [13] analyzes pedestrian interactions globally and highlights the normalcy and rationality of the predicted trajectories regarding social rules; in other words, the model generates more reasonable pathways than existing prediction models. In the meantime, the model resolves the issue of a single trajectory output prediction that differs from reality. However, the features extracted by this model during pooling are the maximum features after maximum pooling, ignoring other feature information that is useful for pedestrian interaction.

Additionally, transformer [14]-based models have drawn a lot of interest. STAR [15] uses a Transformer-based graph convolution mechanism to model intra-graph population interactions. A separate temporal Transformer is used to model the temporal dependencies between graphs, doing away with recursion completely while accounting for the roles and interrelationships of the spatio-temporal dimensions. In terms of applying the underlying framework of this paper—the graph convolutional network (GCN [16])—which expands on the idea of the convolutional neural network(CNN), to graphs, the convolutional operation specified in the graph aggregates the target node’s attributes with those of its neighboring nodes. Social-STGCNN [17] solves the issues of gradient vanishing and cyclic structural error accumulation by extracting spatiotemporal features using graph convolution and TCNs [18]. However, utilizing kernel functions to describe interactions makes adjusting to pedestrians with varying densities challenging. A similar pattern collapse risk exists in Social-BiGAT [19], which combines the game structure of GAN [20], introduces GAT [21] (Graph Attention) to compute collisions between rows, and concentrates on contextual information through a bidirectional structure.

The social-STGCNN paper models pedestrian interactions using a graph representation. It compares aggregation approaches with graph topologies and demonstrates that they offer a more straightforward, intuitive, and effective way to model pedestrian interactions. Thus, we have chosen to adhere to the general framework of Social-STGCNN; nevertheless, we contend that because they established a pedestrian model using an undirected graph and simulated pedestrian interactions using simple nuclear functions, Social-STGCNN did not fully utilize the graph representation. This is insufficient. While Social-STGCNN mitigates gradient vanishing through spatio-temporal graph convolutions, it suffers from three critical limitations: (1) *Undirected Graph Modeling*: Assumes symmetric interactions between pedestrians ($e_{ij}^{t}=e_{ji}^{t}$), contradicting real-world asymmetric avoidance behaviors. (2) *Static Kernel Functions*: Uses fixed-distance kernels to compute interaction forces, failing to adapt to density variations in crowded scenarios. (3) *Single TCN*: Using a traditional single TCN will lead to information loss and limit the prediction accuracy. Traditional TCN is built using MLP and lacks the ability to capture local nonlinear relationships.By performing on a spatio-temporal graph model of the scene, OVK-STGCNN gains more from graph representation.

To achieve the above goals, the following are the primary contributions of this work:

- It demonstrates how pedestrian trajectory prediction has significantly improved thanks to the TCN prediction effect incorporating KANs [22].
- An asymmetric binocular overlapping view method was designed to process the pedestrian coordinate data and better simulate the forces between pedestrians.
- The hotel and eth datasets are used to test this approach, and the lifting results are promising.

Furthermore, we showcase our experimental findings, which include comparing several KANs models in TCN. These experiments provide empirical evidence for the effectiveness of KANs fusion.

## Proposed method

This section first formally presents the problem. Next, we review the OV-SKTGCNN model’s general structure. Then, a TCN structure fused with KAN convolution and a data processing model based on graph structure and field of view domain are presented. Describing the TXP-CNN structure is the last component in producing the prediction findings.

### Problem formulation

Predicting pedestrian positions based on past placements is the aim of pedestrian trajectory prediction. Specifically, given a scene with pedestrians, each pedestrian’s coordinates are observed for a predetermined amount of time, *T*_*obs*_, and the aim is to forecast each pedestrian’s future coordinates from *T*_*obs*_ to *T*_*pred*_. Given a collection of N pedestrians in a scenario,n∈{1,...,N}, and ${tr}_{o}^{n}$ denote their corresponding observed positions. The projected trajectory for a pedestrian n is written as ${tr}_{o}^{n}=\{p_{n}^{t}=(x_{n}^{t},y_{n}^{t})|t\in \{T_{obs},...,T_{pred}\}\}$, where $\left(x_{n}^{t},y_{n}^{t}\right)$ are random variables that describe the probability distribution of pedestrians’ location in 2D space at time *t*.

### Model description

**Fig 1:**
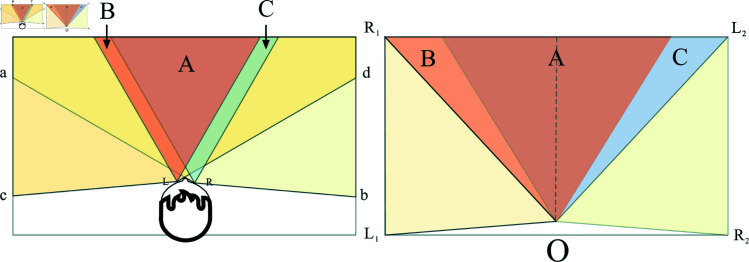
The field of view of a natural human eye is depicted in the left picture, where the best overlapping fields of view for the left and right eyes, respectively, are B, C, and A. Based on the left photo, the right picture has been simplified.

**Fig 2:**
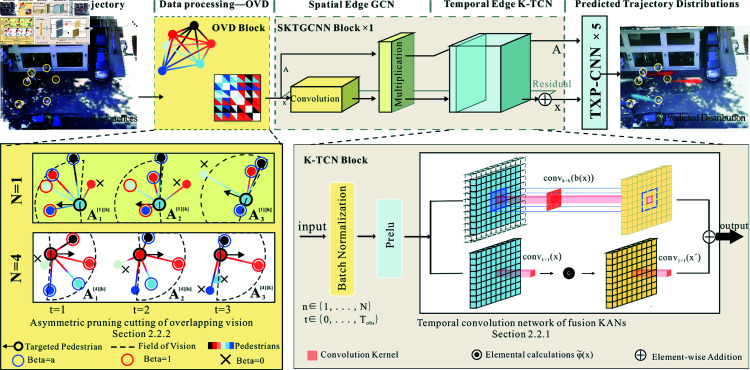
The basic structure of OV-SKTGCNN is depicted in the upper portion of the picture. It primarily comprises three modules: the TXP-CNN prediction output module, the SKTGCNN feature extraction module, and the OVD data processing module. The image on the lower left depicts the exact OVD module implementation procedure, which uses three time steps and two target pedestrians. After integrating Kolmogorov-Arnold Convolutions, the unique structure of K-TCN is shown in the lower right corner.

**Fig 3:**
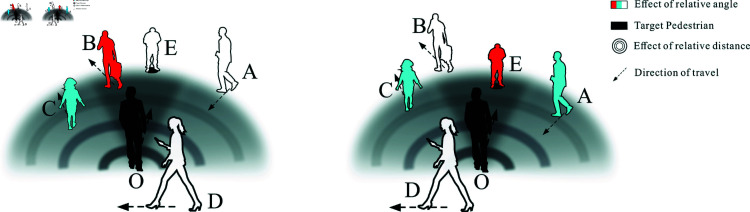
The figure shows how asymmetric pruning is carried out based on the target pedestrian’s relative physical position relative to the surrounding pedestrians. Based on these positional relationships, various influencing factors are determined, ultimately yielding the interaction force between the target pedestrian and other pedestrians.

The OVK-STGCNN framework extends Social-STGCNN by integrating Overlapping Vision-Guided Spatio-Temporal Graph Convolutional Neural Networks (OVK-STGCNN). Unlike traditional undirected graph representations, OVK-STGCNN employs a directed graph to model asymmetric pedestrian interactions, where edge weights $e_{ij}^{t}$ are dynamically adjusted based on binocular overlapping fields of view (Fig 1). The interaction force $e_{ij}^{t}$ is computed as Equation 8. The three primary components of the OV-STKGCNN model (Fig 2) are the Time-Extrapolator Convolution Neural Network (TXP-CNN), the Spatio-Temporal Graph Convolution Neural Network of fusion KANs (SKT-GCNN), and the Data Processing Model based on overlapping visual domains for asymmetric pruning (OVD) (Fig 3). To achieve the impact of actual avoidance, the OVD replicates real vision by carefully dividing the visual field of the human eye. Replace the kernel function with it. To extract features, the SKT-GCNN performs spatio-temporal convolution operations on the graph representation of pedestrian movements. Furthermore, it extracts the time features by combining KANS with time convolution. These attributes constitute a concise representation of the observed pedestrian trajectory history.

#### Temporal convolutional network of fusion KANs.

In the baseline model (Social-STGCNN), STGCNN sequentially extracts spatio-temporal features from processed data. It sends the data explicitly after extracting spatial features using GCN to TCN for temporal feature extraction and complete spatiotemporal feature fusion. The effects of extraction and fusion primarily affect the prediction results of the subsequent TXP-CNN. Still, Social-STGCNN does not use the dilated convolution in the original TCNs [18] for spatio-temporal feature extraction to reason faster and with a smaller number of parameters but only uses the traditional 2D convolution to simulate the one-dimensional features of the time-series data and carry out the feature extraction. However, some global knowledge is lost, as is the accuracy of predictions. To address the issues raised above, the K-TCN network, inspired by the Kolmogorov-Arnold network (KANS), is presented, which enhances prediction accuracy while keeping the number of parameters and inference speed constant.

Instead of using typical TCNs’ dilated convolution to enhance the receptive field and capture temporal dependencies, K-TCN reconstructs the TCN module using the KAN model. MLP is typically used to build CNNs, which are enlarged and optimized. KANs are regarded as a viable alternative to MLP in that the activation function of KANs differs from that of MLP in terms of edges; the activation function of KANs is fixed on points, and because KANs is a univariate function parameterized by a spline curve, it outperforms MLP in terms of accuracy. The first reason is that the spline function can fit arbitrary functions in a grid. KANs use the Grid Extension technique to train a KAN with fewer parameters to control costs and then refine the spline grid to extend it to a KAN with more parameters, eliminating the need to re-train a larger model from scratch. Assume a kth-order B-Spline is utilized to fit a one-dimensional function *f* in a bounded region. The coarse-grained grid has *g*_1_ nodes, and there are *g*_1_ + *k* B-spline basis functions in *g*_1_ + 2*k* nodes after augmentation by kth-order augmentation. The coarse-grained f-functions are represented by a linear combination of the above B-spline basis functions: $f_{coarse}=\sum_{i=k+1}^{g1+2k}c_{i}B_{i}(x).$

Similarly, using a fine-grained grid (g2) representation of the f-function: $f_{fine}=\sum_{j=k+1}^{g2+2k}c_{j}^{\prime }B_{j}^{\prime }(x),$ where the minimum value of $c_{j}^{\prime }$ can be obtained by least-squares de-computing the difference between *f*_*coase*_ and *f*_*fine*_:

$$\{c_{j}^{\prime }\}=\underset{\left\{c_{j}^{\prime }\right\}}{\text{argmin}}{𝔼}_{x\sim p(x)}(f_{\text{coarse}}-f_{\text{fine}}{)}^{2}$$ (1)

Second, because KANs are locally changeable and can avoid the destruction of retained knowledge through the localization of the spline function, they can partially overcome the catastrophic forgetting problem and provide improved sustainable learning. By definition, the spline function is constructed individually on a succession of surrounding intervals (subspaces), and due to its constructive structure, it satisfies specific smoothness constraints at each node. The preceding requirements imply that changing the form of the spline function at one interval does not affect the function’s form at subsequent intervals.

**Fig 4:**
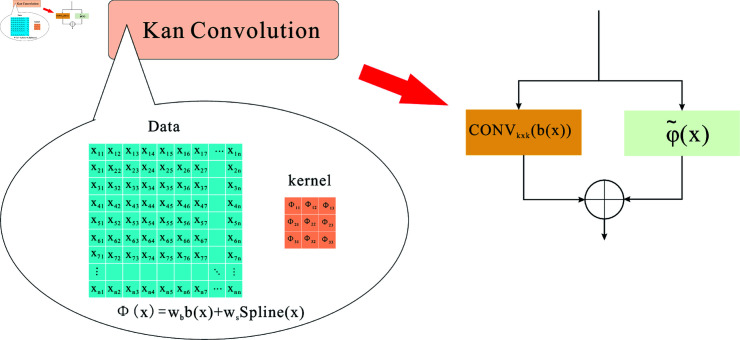
Using an as the Kan convolution of the convolution kernel, one can compute b(x) and Spline (x) separately before fusing and adding them together.

To summarize, we construct the K-TCN module using various related KAN models and then use the Bottleneck Kolmogorov-Arnold Convolutions [23] by selecting the best ones, which is the structure of Kan Convolutions [24] based on the addition of two convolutions, as shown in the bottom-right subplot in Fig 2. Fig 4 depicts the structure of Kan Convolutions, with ϕ(x) as the convolution kernel for window sliding. The complete convolution process is divided into two components, ${conv}_{k\times k(b(x))}$ and $\tilde{\varphi }(x)$. Its primary use is to execute convolutional operations using the Kolmogorov-Arnold network. The primary difference between CNN and KAN convolutions is that the CNN comprises weights. Still, each convolutional KAN element (ϕ) kernel is a learnable nonlinear function based on B-Splines. The KAN convolution kernel travels the data, applying the associated activation function, $\phi_{j}^{i}$, to each pixel individually. The output is thus determined as $\phi_{j}^{i}(a_{l}^{k})$.

$$\phi =w_{b}\cdot b(x)+\tilde{\varphi }(x)$$ (2)

$$\tilde{\varphi }(x)=w_{s}\cdot Spline(x)$$ (3)

$$b(x)=\frac{x}{1+e^{-x}}$$ (4)

Spline(x) is basically parameterized as a linear combination of b-splines:

$$Spline(x)=\sum_{i}c_{i}B_{i}(x)$$ (5)

where *b*(*x*) denotes the basis function applied during the convolution operation (similar to residual linking) to capture nonlinear relationships, while *Spline*(*x*) is parameterized as a linear combination of B-splines, and c is a trainable parameter. Factorization allows better control of the overall magnitude of the activation function.

Bottleneck Kolmogorov-Arnold Convolutions have two more 1 × 1 convolutions than Kan Convolutions. This parametrically efficient architecture can be viewed as a collection of single-layer codecs built by Kan Convolutions to reduce overfitting and memory needs. The decoder’s single layer helps extract significant characteristics from the input, and the residual activation helps maintain the necessary details that may be lost during the input encoding and decoding process.

Experiments evaluating the KANs model revealed that merging Bottleneck Kolmogorov-Arnold Convolution with TCN improves prediction accuracy and outperforms other KANs-related models. However, the existing model’s overall structure is inflexible and requires optimization for the data processing component to collect pedestrian interaction information fully. As a result, we updated the kernel function in the baseline model to better depict the forces between pedestrians using genuine human eyesight.

#### Asymmetric pruning cutting of overlapping vision.

The foundation of the trajectory prediction is still the diagram structure. A collection of created space graphs shows the relative position of pedestrians in a scene at each time step *t* is represented by the variable ${\text{G}}^{\text{t}}$. The graph ${\text{G}}^{\text{t}}$ is defined as $G^{t}=(V^{t},E^{t})$, where the set of vertices of the graph Gt is represented by $V^{T}=V_{i}^{t}|\forall i\in \{1,...,N\}$. The characteristic of $v_{i}^{t}$ is the observed location $\left(x_{i}^{t},y_{i}^{t}\right)$. The edges in graph Gt are denoted by Et, which can be written as $E^{t}=\{e_{ij}^{t}|\forall i,j\in \{1,...,N\}$. $p_{i}^{t}$ is the research object in time step *t*, and $e_{ij}^{t}$ shows how $p_{i}^{t}$ and $p_{j}^{t}$ interact.

**Fig 5:**
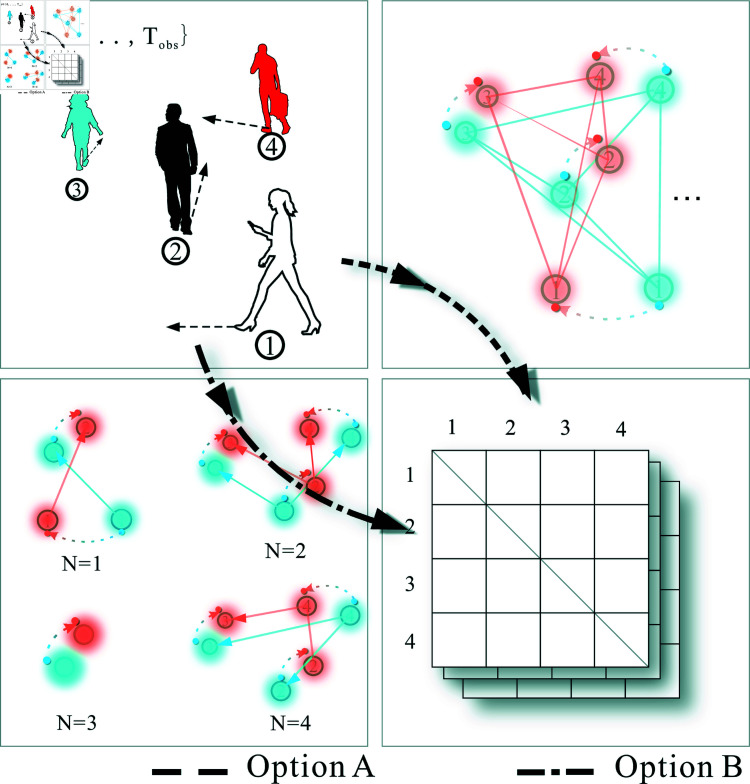
Option A is the traditional step of modeling pedestrians using an undirected graph. Option B is to model each pedestrian using an undirected graph.

While this method lowers the complexity of processing data, reduces redundancy, ensures the integrity of the data, and ensures that no data is lost, it is typically used in many GCN-based prediction models, such as Social-STGCNN, to model pedestrians in a given time step. This means that N pedestrians in time step t are modeled uniformly. The general procedure resembles Fig 5’s Option A. It may seem that two pedestrians shouldn’t interact or should only affect one of them. Still, because of uniform modeling, both pedestrians exert forces on one another, deviating from the prediction trajectory. Unfortunately, this structure cannot simulate a real interaction situation and does not process detailed information. Consequently, as shown in Option B in Fig 5, the data processing approach in this research uses a directed graph to describe each pedestrian in time step *t* independently, discarding the undirected graph structure.

The OVD module (Fig 3) was built by reconstructing the visual field domain, drawing inspiration from [25, 26]. Its purpose is to simulate the genuine visual area of both eyes during a real-world walk and ascertain pedestrian interactions. The corresponding visual fields of the left and right eyes are depicted on the left side of Fig 1. *aRb* includes all regions visible to the right eye. The left eye’s visual field is known as *cLd*. We primarily study area A, the overlapped portion of the ideal comfort zone for one eye and the optimal comfort zone for both eyes. However, the modeling of both eyes is too laborious, and the computation load is too high for trajectory prediction. Thus, make it simpler. The optimal visual region where the eyes overlap is shown by regional A on the right side of Fig 1. The overlapping area of the eyes is ABC. The left eye’s visual limits are designated as $L_{1}OL_{2}$. The region of the right eye is $R_{1}OR_{2}$.

The finest foundation for our forecast is visual experience, as pedestrians’ eyes are their most important sense organs. Among the visual models we developed, pedestrian attention is most likely to be drawn to objects in region A. Thus, the visual domain can be separated into two groups: A and other areas. On the other hand, in different regions, the significant degree must also progressively decrease following the increasing angle. Put differently, when it comes to visible borders, the interaction force between the two sides is extremely little, but it cannot be zero entirely, and the portion that goes beyond the visual area has undergone complete cutting.

As a result, we build pedestrian interactions using the model mentioned above. The individuals *i* and *j*’s distance and angle from one another are given by equation 6 and 7. Based on the location of the previous time $P_{i}^{\left(t-1\right)}$, the pedestrian *i*’s walking direction is determined:

$$D=\|{𝐏}_{i}^{t}-{𝐏}_{j}^{t}{\|}_{2}$$ (6)

$$\theta =\arccos\frac{\left({𝐏}_{i}^{t}-{𝐏}_{i}^{t-1})\cdot ({𝐏}_{i}^{t}-{𝐏}_{j}^{t}\right)}{\left\|{𝐏}_{i}^{t}-{𝐏}_{i}^{t-1}\|\|{𝐏}_{j}^{t}-{𝐏}_{i}^{t}\right\|}$$ (7)

where *D* is the pedestrians’ joint Euclidean distance at the same time step. θ is the angle formed between the walking direction of pedestrian *i* and pedestrian *j*.

Created a model that describes the interaction relationship based on the calculated pedestrians’ relative physical locations:

$$e_{ij}^{t}=\beta \left(e^{r-D}+\varepsilon \right)$$ (8)

where *r* is the minimum rejection distance, β is the angle influence factor, and $e_{ij}^{t}$ is the force between pedestrians i and *j* at time step *t*. To replicate the stochastic nature of human attention, ε is a tiny random variable that has been included.

$$\beta =\left\{ \begin{array}{cc} 0, & D>r\text{ or }\theta \geq MAX/2\\ e^{-x^{2}/(2\sigma^{2})}, & MIN/2<\theta <MAX/2\\ 1, & 0\leq \theta <MIN/2 \end{array}\right.$$ (9)

where *r* is the minimum repulsive distance, *MAX* is the maximum range of binocular vision. *MIN* is the angle at which the visual fields of both eyes overlap

It is necessary to compute the angle impact factor β based on the current mutual position (Eq 9). If it goes beyond what is visible, the branch-cutting strip is used till zero. It is directly to the maximum value of 1 if it is in the overlapping area A; else, its value is represented by the normalized Gaussian distribution with μ 0.

The minimum repulsive distance (*r*) (Eq 8) is among the OVD module’s above parameters that we should pay particular attention to and test since it affects the prediction effect for varying human-pedestrian densities. It determines how realistic our constructed OVD is in modeling interactions between pedestrians.

**Fig 6:**
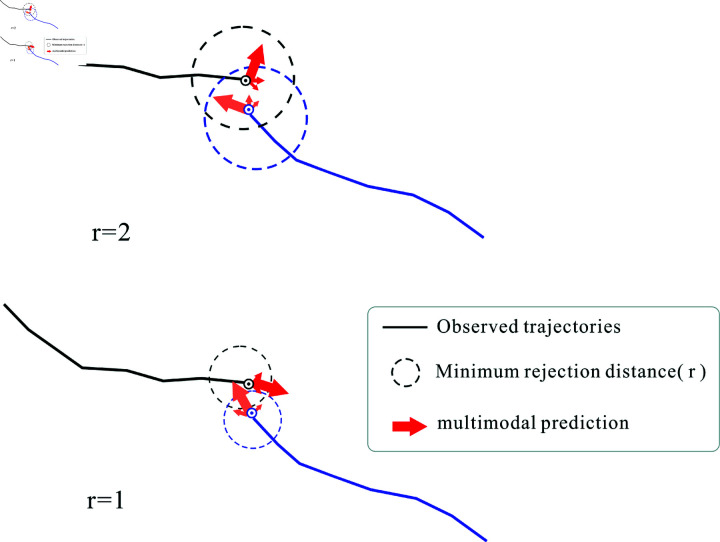
Multimodal prediction of pedestrians during encounters when r = 2 and r = 1.

The pedestrian’s multimodal trajectory presents several options, but the probability of reversing course to avoid a collision is highest at *r* = 1, as illustrated in Fig 6. Both pedestrians appear to be within the sphere of influence at this point. However, at *r* = 2, both pedestrians appear in the same place and do not interact with one another. As a result, they are most likely to walk in the original direction, which increases the likelihood of collisions and results in a significant deviation between the true and predicted trajectories, which is not good for prediction results. Similarly, when the minimum rejection distance decreases, the pedestrian’s movement direction seems to shift excessively or the time point sooner, which is detrimental to precise prediction.

#### OVD module analysis.

The relationship between pedestrian O (black) and other pedestrians is depicted in Fig 3 over a while, comprising two time steps, *t* = 1 and *t* = 2. The pedestrian’s total field of vision is the entire sector area. The darkest area is the overlapped region where the research subject will most likely concentrate. The arrow indicates the pedestrian’s direction of travel. Pedestrian E has not moved. B first appears in the overlapped region at *t* = 1. B’s mark is red because, besides determining the relative distance between the two parties, B’s angle influence factor (β) is 1. Since Pedestrian C is in the color gradient region, the angle must be considered while calculating the related β value. It has a blue mark. Pedestrians E, A, and D do not impact research subject O; they appear beyond the field of vision, with a β of 0 and white markings.

However, at *t* = 2, the figure shows that B has moved out of O’s field of view and that E and A have entered O’s impact region due to their walking. As a result, the matching signs for B, E, and A will also change. The interaction force will be redefined in light of shifting pedestrian conditions in the area.

## Experiments and discussion

This part first covers the model configuration and training setup, followed by the dataset utilized, evaluation measures, and implementation specifics. Next, we use the dataset to examine the findings of the comparison between OV-SKTGCNNN and other models. Lastly, we finish a fusion comparison experiment of models linked to KANS and three ablation investigations for both modules.

### Data

Two publicly accessible datasets are frequently utilized in literature: ETH [27] and UCY [28]. They comprise five scenes: two from ETH (called ETH and Hotel) and three from UCY (called Univ, Zara1, and Zara2). They include annotations for pedestrian positions every 0.4 seconds, totaling over 1600 pedestrian trajectories. We employed the same "leave-one-out" tactic as Social-LSTM and Social-STGCNN. Four datasets were used to train and validate the model, while the remaining datasets were used for testing. We went through this process again for all five datasets. The training and testing process for the other benchmark methods utilized for comparison was the same. In the evaluation process, the model tracks the path of 8 frames and forecasts the path of the following 12.

### Metrics

The predicted trajectories are evaluated using two metrics: the Average Displacement Error (ADE [27]), given in Eq 10, and the Final Displacement Error (FDE [11]), defined in Eq 11. Intuitively, ADE assesses average prediction performance along the trajectory, whereas FDE solely analyzes prediction precision at the endpoints.

$$ADE=\frac{\sum_{n\in N}\sum_{t\in T_{\text{obs}}}\|{\hat{p}}_{t}^{n}-p_{t}^{n}{\|}_{2}}{N\times T_{\text{pred}}}$$ (10)

$$FDE=\frac{\sum_{n\in N}\|{\hat{p}}_{t}^{n}-p_{t}^{n}{\|}_{2}}{N},\;t=T_{\text{pred}}$$ (11)

### Model configuration and training setup

OV-SKTGCNN has a similar overall structure as Social-STGCNN. It comprises a series of SKT-GCNN layers, followed by TXP-CNN layers. According to our findings in Table 4, the optimum model consists of one SKTGCNN layer and five TXP-CNN layers.

**Fig 7:**
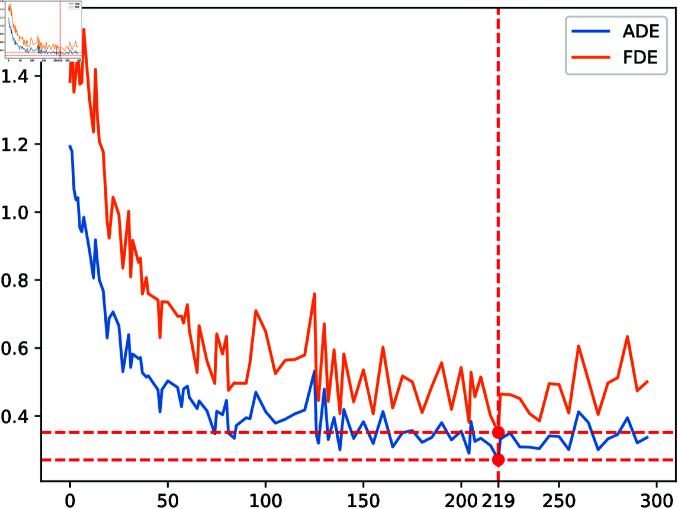
The prediction result is displayed on the hotel dataset, which includes the training procedure when the loss function is decreased and the model saved with training rounds as a multiple of five.

We also fixed the training batch size to 128 and trained the model via Stochastic Gradient Descent (SGD). However, we increased the number of training sessions to 300 times since we discovered that while the loss value of some rounds of training models is not the lowest, the evaluation index of this model may be the best (Fig 7). Model performance was evaluated at every 10 epochs using validation ADE/FDE, where early stopping occurs if there is no improvement for 20 epochs, and the learning rate is reduced by 50% if the training loss plateaus for 15 epochs. We employed two distinct methods to save models during training to avoid missing the excellent model. In Table 3, we examine the settings of the OVD module, including the minimum exclusion distance participation and the Gauss distribution σ, for a comparative experiment. Finally, *r* is set to 2, and σ equals 30.

### Comparative experimental analysis

We compared our OV-SKTGCNN to the following cutting-edge techniques: CGNS [29], ST-GAT [30], STSGN [31, 31], GAT [21], Social-BiGAT [19], Social-GAN [13], Social-LSTM [11], SRASIGAN [32], WR-SRPG [33], Social-STGCNN [17], Social-STGCNN+SEAD [34], PTP-STGCNN [35] , STT [36] and High-order GCN [37]. Overall, OV-SKTGCNN surpasses all previous techniques on both metrics (0.36 and 0.58, respectively). In Table 1, using performance metrics ADE and FDE in meter scale, we present the comparison results between OV-SKTGCNN and the state-of-the-art works. The experimental results demonstrate the competitiveness and good performance of the OV-SKTGCNN approach when compared to other models. In the experimental results with ADE as the evaluation metric, the models SRASIGAN, WR-SRPG, STT, and High-order GCN have better prediction results than OV-SKTGCNN in several datasets; however, the results of OV-SKTGCNN are better in FDE experiments, indicating that there is still room for improvement in ADE evaluation metrics. ADE considers the overall quality of the anticipated trajectory, whereas FDE analyzes the trajectory’s ultimate point. As a result, OV-SKTGCNN is more biased toward predicting final coordinates, which can help to lessen the likelihood of collisions. When comparing multiple datasets, the prediction results of OV-SKTGCNN on the UCY dataset are superior to ETH. The UCY dataset has a large and dense population of pedestrians, and the distance threshold used there is appropriate for the scene, which makes it easier to obtain the forces between pedestrians.

**Table 1:** The ADE/FDE metrics for various approaches compared to OV-SKTGCNN are displayed. The remaining models utilized the best 20 samples for evaluation. Every model predicts the following 12 frames based on 8 input frames. OV-SKTGCNN has the best average error on both ADE and FDE criteria. The lower, the better.

Models	Datasets					
——	ETH	HOTEL	UNIV	ZARA1	ZARA2	AVG
*CGNS* [29]	0.62/1.40	0.70/0.93	0.48/1.22	0.32/0.59	0.35/0.71	0.49/0.97
*ST*–*GAT* [30]	0.90/1.96	0.41/0.83	0.57/1.19	0.41/0.89	0.32/0.70	0.52/1.11
*STSGN* [31]	0.75/1.63	0.63/1.01	0.48/1.08	0.30/0.65	**0.26**/0.57	0.48/0.99
*GAT* [21]	0.68/1.29	0.68/1.40	0.57/1.29	0.29/0.60	0.37/0.75	0.52/1.07
*Social*–*BiGAT* [19]	0.69/1.29	0.49/1.01	0.55/1.32	0.30/0.62	0.36/0.75	0.48/1.00
*Social*–*GAN* [13]	0.87/1.62	0.67/1.37	0.76/1.52	0.35/0.68	0.42/0.84	0.61/1.21
*Social*–*LSTM* [11]	1.09/2.35	0.79/1.76	0.67/1.40	0.47/1.00	0.56/1.17	0.72/1.54
*SRASIGAN* [32]	0.56/1.17	0.41/0.81	0.40/0.98	**0.28**/0.68	0.29/0.66	0.38/0.86
*WR*–*SRPG* [33]	0.65/1.08	**0.18**/0.37	0.39/0.71	0.33/0.68	**0.26**/0.45	**0.36**/0.65
*Social*–*STGCNN* [17]	0.64/1.11	0.49/0.85	0.44/0.79	0.34/0.53	0.30/0.48	0.44/0.75
*Social*−*STGCNN* + *SEAD* [34]	0.66/1.12	0.36/0.58	**0.33/0.51**	0.29/0.47	0.46/0.85	0.42/0.71
*PTP*–*STGCNN* [35]	0.63/1.04	0.34/0.45	0.48/0.87	0.37/0.61	0.30/0.46	0.42/0.68
*STT* [36]	**0.54**/1.10	0.24/0.46	0.57/1.15	0.45/0.94	0.36/0.77	0.43/0.88
*High*–*orderGCN* [37]	**0.54**/1.09	0.24/0.44	0.41/0.89	0.32/0.70	0.53/1.14	0.41/0.85
*OV*–*SKTGCNN*	0.58/**0.98**	0.27/**0.35**	0.38/0.69	0.31/**0.50**	**0.26/0.42**	**0.36/0.58**

The last row is the experimental data for the model(OV-SKTGCNN) in this paper.

**Fig 8:**
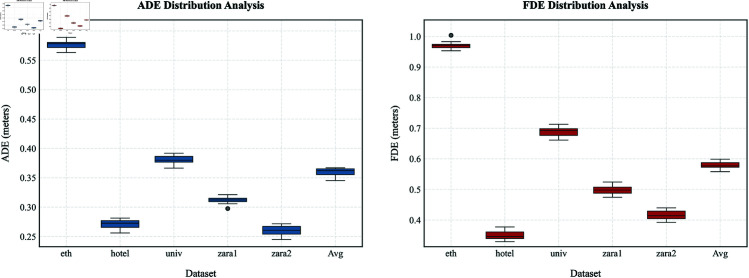
The narrowest ADE interquartile range (IQR: 0.28–0.32 m for zara1) reflects robustness in dense crowd scenarios. Outliers in univ suggest challenges in complex crossing path modeling. FDE IQR for Avg (0.57–0.59 m) shows 21.3% lower dispersion compared to single-scenario extremes, validating cross-scenario training’s regularization effect.

In contrast, the ETH dataset has a small number of relatively dispersed pedestrians, which makes it more challenging to determine whether the target object has been avoided. Furthermore, the parameter selections may impact the model’s prediction performance when trimming utilizing overlapping visual domains. The parameters don’t undergo any additional processing to allow them to adjust to various data densities.However, since repeated experimental trials introduce variability in the data that could compromise the reliability of the model’s predictive performance, Fig 8 displays the error boxplots for the ADE and FDE metrics derived from multiple training and testing iterations, with the figure caption providing detailed explanations of the visualized results.

### Ablation study

We carried out several ablation investigations to examine the effects of the different parts and parameter settings on the OV-SKTGCNN’s performance.

#### OVD and K-TCN.

The findings of the ablation tests with OVD and K-TCN are summarized in Table 2, which also provides additional validation of the effects of OVD and K-TCN on the overall prediction outcomes. 1 the average prediction result is ADE:0.37, FDE:0.64, and only the K-TCN module is introduced. Through experimental data and combined with the above (section 1 *Temporal convolutional network of fusion KANs*), it can be seen that K-TCN can use the characteristics of KANs that can fit arbitrary functions, local variability, and the single-layer “encoder-decoder” of Bottleneck Kolmogorov-Arnold Convolutions to provide continuous learning of temporal features, and pay more attention to necessary details that may be lost while capturing global information, thereby improving the overall prediction accuracy. In 2, ADE:0.38 and FDE:0.61 are the only changes to the data processing method and the OVD module. Because it has two field-of-view settings, the OVD module (section 1 *Asymmetric pruning cutting of overlapping vision*) has higher FDE assessment metrics in ZARA1 and ZARA2. Parameter settings primarily work with datasets that have a lot of pedestrian activity. Nevertheless, The current trials did not allow for adaptive adjustments of these parameters in response to variations in data density. Because it can collect specific information that could otherwise be lost, the K-TCN is better suited for low-density data.

**Table 2:** The outcomes of ablation experiments conducted on K-TCN and OVD models are presented using ADE and FDE as metrics and ETH and HOTEL as datasets.

Models	Datasets					
——	ETH	HOTEL	UNIV	ZARA1	ZARA2	AVG
*Social*–*STGCNN*	0.64/1.11	0.49/0.85	0.44/0.79	0.34/0.53	0.30/0.48	0.44/0.75
1	0.62/1.06	**0.27**/0.45	0.40/0.71	0.33/0.55	0.27/0.45	0.37/0.64
2	0.64/1.08	0.30/0.39	0.39/0.70	0.32/0.50	0.27/**0.42**	0.38/0.61
*OV*–*SKTGCNN*	**0.58/0.98**	**0.27/0.35**	**0.38/0.69**	**0.31/0.50**	**0.26/0.42**	**0.36/0.58**

1: This model contains only K-TCN and does not include OVD. 2: This model contains only OVD and does not include K-TCN.

#### Minimum rejection distance(*r*) and σ.

As mentioned in section 1 *Asymmetric pruning cutting of overlapping vision*, the minimum exclusion distance, also known as the radius of the influence area or the social distance between people, influences a pedestrian’s future trajectory when nearby pedestrians appear within the target object’s influence area. The setting of σ determines how much the interaction force between pedestrians is reduced when pedestrians appear within the area of influence of the target object but not within the overlap.

**Fig 9:**
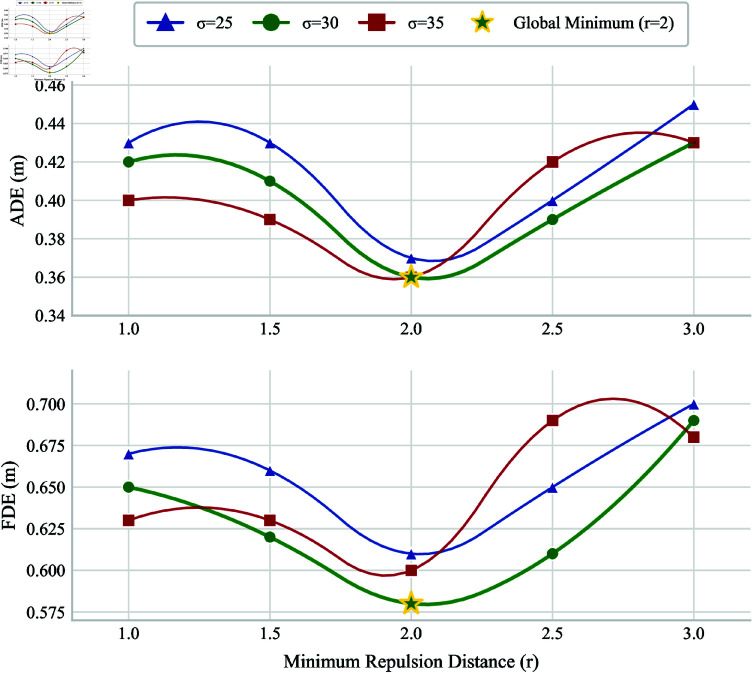
The global minimum (pentagram at *r* = 2.0m) achieves optimal balance between ADE and FDE. Contour lines for different scenario parameters (σ = 25,30,35) confirm the robustness of this solution, establishing *r* = 1.0m as a critical configuration for model generalization.

Through grid search experiments, we empirically determined the optimal parameters: minimum repulsion distance *r* = 2 and Gaussian distribution σ=30 (Fig 9). These values balanced collision avoidance accuracy and computational efficiency across varying pedestrian densities.

It is important to note that *r* = 2 effectively reduces collisions in UCY, but may lead to excessive avoidance in ETH, so a trade-off in parameter selection is required.

#### Layers of SKTGCNN and TXP-CNN.

We need to re-experiment with the layer settings of SKTGCNN and TXP-CNN because we have introduced OVD and K-TCN. The values of STGCNN and TXP-CNN in Social-STGCNN are 1 and 5, respectively. Table 3’s experimental findings demonstrate that the number of layers is still best predicted to be between 1 and 5.

**Table 3:** Performance of SKTGCNN and TXP-CNN with varying layers

	1	3	5	7
1	0.38/0.63	0.42/0.66	0.43/0.69	0.40/0.72
3	0.40/0.61	0.45/0.70	0.44/0.71	0.44/0.72
5	**0.36/0.58**	0.47/0.74	0.46/0.76	0.50/0.80
7	0.45/0.95	0.59/0.11	0.65/1.05	0.69/1.19

Note: The number of SKTGCNN layers of the data in the row. Columns are the number of layers of TXP-CNN.

### Experiment evaluating the KANs model

In this section, we combine various KAN convolutions in the SKTCNN module with KAN Linear and compare their relative evaluation indicators, such as Fast Kan Convolutions [38], Wav Kan Convolutions [40], Bottleneck Kolmogorov-Arnold Convolutions [23], Kan Convolutions [24], Relu Kan Convolutions [39], and Kan Linear [22].

The experimental results in Table 4 demonstrate that the fusion of KANs is more appropriate for models with higher data density and can better extract spatiotemporal features. After fusing all of the aforementioned KANs-related models with TCN, the prediction effect in HOTEL is essentially better than the baseline model when compared with the ETH dataset. Except for Bottleneck Kolmogorov-Arnold Convolutions, the evaluation values of the fusion models in ETH are significantly higher than those of the original models, and their prediction effects exhibit varying degrees of deterioration. The 1×1 convolutions, which are equivalent to two single-layer encoders and decoders in the fused Bottleneck Kolmogorov-Arnold Convolutions model, aid in the extraction of significant features from the input and preserve any subtleties that would be lost during the convolution process. As a result, the fusion of this convolution may significantly enhance the prediction effect and solve the issue of not getting all characteristics because of the limited amount of data.

**Table 4:** Experimental results of fusion of TCNs by different KANs models.

Fusion Module	Datasets					
——	ETH	HOTEL	UNIV	ZARA1	ZARA2	AVG
*KANlinear* [22]	0.70/1.15	0.31/0.44	0.40/0.71	0.35/0.57	0.28/0.46	0.40/0.66
*FastKanConvolutions* [38]	0.78/1.30	0.45/0.70	0.39/**0.68**	0.33/0.54	0.29/0.46	0.44/0.73
*BottleneckKolmogorov*–*ArnoldConvolutions* [23]	**0.62/1.06**	**0.27**/0.45	0.40/0.71	0.33/0.55	**0.27**/0.45	**0.37/0.64**
*KanConvolutions* [24]	0.74/1.44	0.39/0.59	0.40/0.72	0.33/**0.52**	0.29/0.47	0.43/0.75
*ReluKanConvolutions* [39]	0.74/1.24	**0.27/0.36**	0.40/0.72	0.33/0.54	0.29/0.51	0.40/0.67
*WavKANConvolutions* [40].	0.82/1.41	0.32/0.48	0.37/0.69	0.32/0.52	0.27/0.43	0.42/0.71

1: This model contains only K-TCN and does not include OVD. 2: This model contains only OVD and does not include K-TCN.

**Fig 10:**
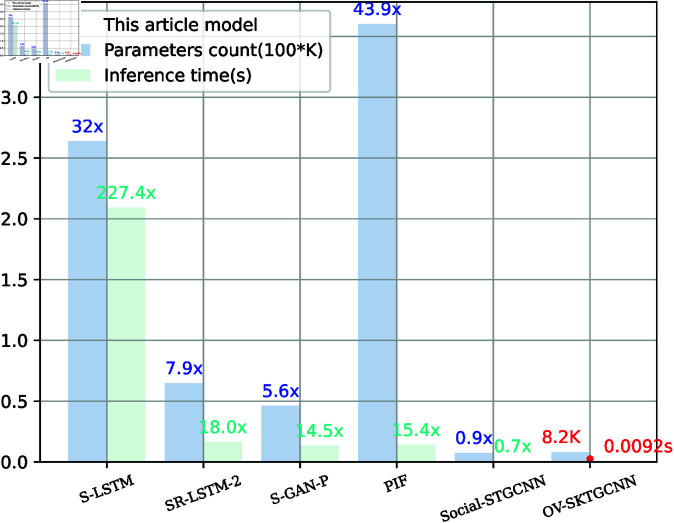
Models other than ours in terms of parameter size and inference time. Lower is preferable. The Nvidia GTX1050Ti GPU was used to benchmark the models. An inference time is calculated by averaging multiple individual inference steps. The number of times our model outperforms other models is indicated by the ^′^×^′^.

We also studied SKTGCNN after incorporating Bottleneck Kolmogorov-Ratnold Convolutions for parameter and reasoning time in Fig 10. Although SKTCNN parameters are approximately 0.6K higher than STGCNN’s, they are still many times greater than other models. As a result, we could deduce that SKTCNN uses a smaller parameter in exchange for more accuracy.

### Angle of overlapping visual areas

**Fig 11:**
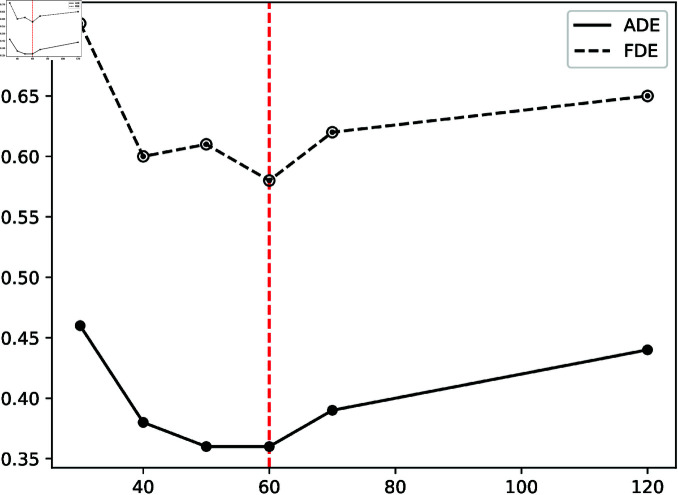
Prediction results for multiple overlapping visual area angles (30,40,50,60,70,120).

The pedestrian’s impact on the target pedestrians in the OVD module is greatest when nearby pedestrians are at the same distance and in the overlapping visual region of the target pedestrians. Fig 11 compares the prediction effects of several angles and finds that the overlapping area’s final angle is 60 degrees.

### Qualitative analysis

**Fig 12:**
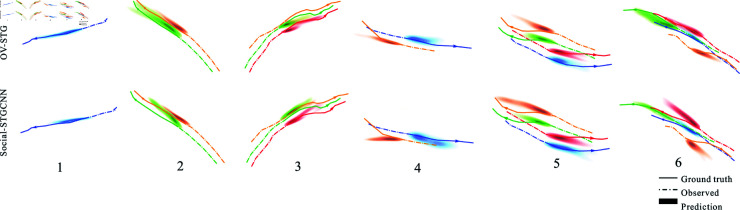
OV-SKTGCNN qualitative analysis. For the comparison, Social-STGCNN is used as the reference point. The datasets used for the illustration scenes are ETH and UCY. Numerous scenarios are displayed: A solitary pedestrian (1), two or three people walking in parallel (2), three people walking in parallel (3), two people meeting from the opposite direction (4), multiple people meeting in parallel from different dir+ections (5), and one person meeting another group of pedestrians from various directions (6) are examples of pedestrian movements. The color density represents the expected trajectory distribution for each instance, while the dashed line represents the actual trajectory that the pedestrians are taking.

**Fig 13:**
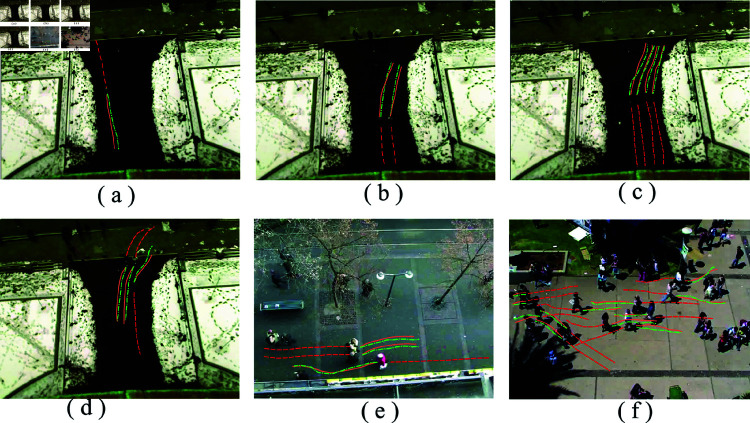
Single-orbit prediction for various scenarios. (**a**) is projected for individual pedestrians. (**b**) is predicted for two people in parallel, and (**c**) for multiple people in parallel. (**d**) is avoiding roundabouts. (**e**) is multiple avoidance. (**f**) is a multi-person scenario.

According to the quantitative analysis section (Figs 12 and 13), OV-SKTGCNN performs better on ADE/FDE criteria than Social-STGCNN. We now perform a qualitative analysis of how OV-SKTGCNN perceives and accounts for pedestrian social interactions when forecasting distributions. We demonstrate scenarios where OV-SKTGCNN accurately predicts the outcome of pedestrian meetings, preserves parallel walking, and successfully predicts collision-free trajectories between walkers approaching from various angles.

#### Parallel walking.

Two and three pedestrians are walking in parallel in scenarios two and three in Fig 12. Individuals walking in parallel are typically closely linked to one another, and their forward momentum will be maintained. OV-SKTGCNN and Social-STGCNN forecasts indicate that these two pedestrians will continue to stroll side by side. In contrast to Social-STGCNN’s divergence, the predicted density of OV-SKTGCNN closely resembles the ground truth trajectory. In scenario 2, two individuals stroll beside each other. Their ground truth trajectory is almost straight, while the OV-SKTGCNN forecast has a minor divergence and is closer to the genuine trajectory. In scenario 3, several persons are walking next to each other. Because of the multiple turns in their trajectory, the OV-SKTGCNN prediction region is near the position following the turn.

#### Collision avoidance.

In Fig 7, two or more pedestrians travel in opposing directions in scenarios 4 and 5. If they keep moving forward, they can crash into each other. Two pedestrians are moving in the opposite direction in scenario 4. We note that the trajectories in the OV-SKTGCNN forecast are somewhat modified to avoid collisions and match the observed pedestrian velocity well. Consequently, OV-SKTGCNN matches ground truth more accurately. The prediction results for preventing a collision when several people meet simultaneously are displayed in scenario 5.

## Conclusion

This work proposes the OV-SKTGCNN model for pedestrian trajectory prediction. This study introduces the K-TCN for time modeling and constructs the OVD module to implement part of pedestrian modeling to increase the accuracy of the model prediction. The ETH and UCY datasets were used for ablation experiments and comparison studies. The findings demonstrate that, in comparison to other models, OV-SKTGCNN has fewer errors in the average evaluation indicators of ADE and FDE. In terms of predicting pedestrian trajectories, our approach is competitive. It is evident from qualitative analysis that the anticipated and socially acceptable trajectory’s probability distributions match the actual circumstances. Nevertheless, there are certain flaws in the OVD model parameter setup, and it cannot handle data sets with varying densities adaptively. So, the next effort will investigate how to choose two parameters for various data sets optimally, minimizing the errors of ADE and FDE without compromising the number of model parameters or the real-time performance. While OV-SKTGCNN achieves state-of-the-art performance on ETH/UCY, its fixed parameters (*r* = 2, σ=30) may limit generalizability to extremely dense crowds (e.g., > 5 pedestrians/${\text{m}}^{2}$). Future work will explore adaptive parameter tuning via reinforcement learning.
